# Assessment of different genotyping markers and algorithms for distinguishing *Plasmodium falciparum* recrudescence from reinfection in Uganda

**DOI:** 10.1038/s41598-025-88892-7

**Published:** 2025-02-05

**Authors:** Alex Mwesigwa, Monica Golumbeanu, Sam Jones, Sara L. Cantoreggi, Benson Musinguzi, Joaniter I. Nankabirwa, Everd Maniple Bikaitwoha, Joan N Kalyango, Charles Karamagi, Mateusz Plucinski, Samuel L. Nsobya, Christian Nsanzabana, Pauline Byakika-Kibwika

**Affiliations:** 1 Clinical Epidemiology Unit, School of Medicine, College of Health Sciences, Makerere University, P. O. Box 7072, Kampala, Uganda; 2 Department of Microbiology and Immunology, School of Medicine, Kabale University, P. O Box 314, Kabale, Uganda; 3 Swiss Tropical and Public Health Institute, Allschwil, Switzerland; 4 University of Basel, Basel, Switzerland; 5 MMV Medicines for Malaria Venture, Geneva, Switzerland; 6 Departent of Medical Laboratory Science, Faculty of Health Sciences, Muni University, P.O Box 725, Arua, Uganda; 7 Infectious Diseases Research Collaboration, College of Health Sciences, Makerere University, P.O. Box 7072, Kampala, Uganda; 8 Department of Community Health, School of Medicine, Kabale University, P. O Box 314, Kabale, Uganda; 9 U.S. President’s Malaria Initiative, Centers for Disease Control and Prevention, Atlanta, GA 30345 USA; 10 Mbarara University of Science and Technology, Mbarara, Uganda

**Keywords:** *Plasmodium falciparum*, Recrudescence, Reinfection, Microsatellites, *msp-1*, *msp-2*, Antimalarial drug, Molecular biology, Health care, Medical research

## Abstract

Antimalarial therapeutic efficacy studies are vital for monitoring drug efficacy in malaria-endemic regions. The WHO recommends genotyping polymorphic markers including *msp-1*, *msp-2*, and *glurp* for distinguishing recrudescences from reinfections. Recently, WHO proposed replacing *glurp* with microsatellites (Poly-α, PfPK2, TA1). However, suitable combinations with *msp-1* and *msp-2*, as well as the performance of different algorithms for classifying recrudescence, have not been systematically assessed. This study investigated various microsatellites alongside *msp-1* and *msp-2* for molecular correction and compared different genotyping algorithms across three sites in Uganda. Microsatellites 313, Poly-α, and 383 exhibited the highest diversity, while PfPK2 and Poly-α revealed elevated multiplicity of infection (MOI) across all sites. The 3/3 match-counting algorithm classified significantly fewer recrudescences than both the ≥ 2/3 and Bayesian algorithms at probability cutoffs of ≥ 0.7 and ≥ 0.8 (*P* < 0.05). The *msp-1*/*msp-2*/2490 combination identified more recrudescences using the ≥ 2/3 and 3/3 algorithms in the artemether-lumefantrine (AL) treatment arm, while *msp-1*/*msp-2*/*glurp* combination classified more cases of recrudescence using the ≥ 2/3 in the dihydroartemisinin-piperaquine (DP) arm. Microsatellites PfPK2 and Poly-α, potentially sensitive to detecting minority clones, are promising replacements for *glurp*. Discrepancies in recrudescence classification between match-counting and Bayesian algorithms highlight the need for standardized PCR correction practices.

**Supplementary Information:**

The online version contains supplementary material available at 10.1038/s41598-025-88892-7.

## Introduction

Malaria remains a major global health challenge, with *Plasmodium falciparum* (*P. falciparum*) responsible for the majority of malaria-related deaths worldwide^1,2^. Antimalarial drug resistance, especially in *P. falciparum*, poses a significant threat to malaria control efforts, making rigorous monitoring of drug efficacy crucial through clinical trials^3,4^. Consequently, malaria-endemic countries are advised to conduct therapeutic efficacy studies (TES) at least every two years^5,6^.

A key challenge in TES is distinguishing recrudescence (the recurrence of asexual parasitemia caused by the same genotype(s) that caused the original infection) from reinfection (a new infection after the primary one) in patients with recurrent malaria^6–8^. The WHO recommends using genotyping to distinguish recrudescence from reinfection through molecular correction, and suggests changing first-line antimalarial therapy if the molecular-corrected failure rate exceeds 10%^9^. The ability to accurately distinguish recrudescence from reinfection depends on the sensitivity of the genotyping methods, the genetic diversity of the markers used, and the classification algorithms applied^10^. High-throughput methods like amplicon sequencing can overcome the limitations of length-polymorphic markers, such as glutamate-rich protein (*glurp*), which may fail to detect minority clones, resulting in more accurate differentiation between recrudescence and reinfection^11^.

The WHO 2008 guidelines recommended the use of three polymorphic genetic markers for molecular correction: merozoite surface protein 1 (*msp-1*), merozoite surface protein 2 (*msp-2*), and *glurp*^12^. However, *glurp* has been found to have limitations in detecting minority clones in polyclonal infections^13^. Recently, the WHO recommended replacing *glurp* with polymorphic microsatellites, including Poly-α, PfPK2, and TA1, to address these limitations^10^. These highly polymorphic markers are identified as cost-effective and unbiased tools for use in TES in malaria-endemic areas^14^. The WHO also recommended the use of a 3/3 match-counting algorithm, which classifies a recurrent malaria infection as recrudescence if one or more alleles are present at all three genotyped markers on both day 0 (the day of the primary infection) and the day of the recurrent infection^10^. Nevertheless, the WHO has emphasized the importance of evaluating additional algorithms, such as the ≥ 2/3 match-counting algorithm, which classifies a recurrent infection as recrudescence if one or more alleles are found in at least two of the three genotyped markers on both day 0 and the recurrent infection day^10^. Another approach is a probabilistic method using Bayesian statistics to quantify the probability that an infection is either a recrudescence or a reinfection^10,15^.

The choice of genotyping methods for molecular correction is influenced by malaria transmission intensity. In high-transmission areas, robust microsatellite markers, including Poly-α, TA1, TA109, and PfPK2, are preferred due to their cost-effective sensitivity and ability to detect greater genetic diversity within parasite populations. These markers can provide more accurate results and reduce the need for follow-up studies or interventions^14^. In contrast, regions with lower transmission intensities may benefit from simpler markers like *msp-1*, *msp-2*, and *glurp*^10^. According to the 2018–2019 malaria indicator survey, Uganda experiences perennial, high-intensity malaria transmission, with the highest parasite prevalence rates observed in the Karamoja, West Nile, and Busoga subregions, which have prevalence rates of 34%, 22%, 21%, and 13%, respectively, among children aged 0–59 months^16^. This high transmission intensity significantly influences the selection of genotyping markers, with complex markers such as microsatellites being more suitable for high-transmission settings, where greater parasite genetic diversity is observed.

To our knowledge, microsatellites suitable for molecular correction in combination with *msp-1* and *msp-2* have not been systematically evaluated across Uganda’s malaria-endemic regions. Additionally, limited evidence exists regarding the performance of match-counting and Bayesian algorithms in distinguishing recrudescence from reinfection in regions with high *P. falciparum* transmission intensity. Therefore, this study focused specifically on high-transmission settings in Uganda, aiming to identify microsatellite markers that could replace *glurp* and, using various alternative marker combinations, assessed the performance of three algorithms (the 3/3 match-counting, ≥ 2/3 match-counting, and Bayesian approaches) in distinguishing *P. falciparum* recrudescence from reinfection.

## Results

### Diversity of the genotyped markers across three malaria-endemic sites in Uganda

The genotyping results revealed that 174 (98.7%) of the 179 paired samples were successfully processed (Supplementary Table S1). The genetic markers K1, MAD20, RO33, along with the 3D7 and FC27 allelic variants, correspond to the *msp-1* and *msp-2* genes, respectively. The overall mean expected heterozygosity (H_e_) for *P. falciparum* was 0.84 ± 0.12 in Aduku, 0.85 ± 0.12 in Arua, and 0.87 ± 0.09 in Masafu. For *msp-1*, the K1 allelic family exhibited the highest H_e_ values, with 0.88 in Aduku, 0.93 in Arua, and 0.92 in Masafu. For *msp-2*, the 3D7 allelic family displayed the highest H_e_ values, with 0.94 in both Aduku and Arua, and 0.95 in Masafu. The microsatellite marker 313 showed consistent H_e_ values of 0.95 across all sites. The Poly-α marker demonstrated mean expected H_e_ values of 0.92 in Aduku, 0.91 in Arua, and 0.94 in Masafu. Additionally, the overall mean effective number of alleles (N_e_) was 7.61 ± 3.66 in Aduku, 8.77 ± 4.04 in Arua, and 9.24 ± 4.62 in Masafu (Table 1). These findings indicate a relatively high level of genetic diversity across the study sites.

**Table 1:** Mean expected heterozygosity (H_e_) and number of effective alleles (N_e_) of the genotyped markers.

Marker	Aduku		Arua		Masafu	
	H_e_	N_e_	H_e_	N_e_	H_e_	N_e_
K1	0.88	7.45	0.93	12	0.92	10.84
MAD20	0.89	6.67	0.93	11.59	0.9	8.29
RO33	0.63	2.56	0.56	2.21	0.68	2.99
IC/3D7	0.94	12.63	0.94	14.71	0.95	16.26
FC 27	0.87	6.92	0.87	6.92	0.85	6.23
*g*lurp	0.86	6.44	0.89	8.28	0.92	11.71
313	0.95	15.47	0.95	15.72	0.95	17.56
383	0.9	8.97	0.88	7.63	0.85	6.49
Poly-α	0.92	10.47	0.91	9.98	0.94	14.02
TA1	0.86	6.59	0.89	7.63	0.89	8.56
PfPK2	0.87	7.18	0.87	7.15	0.85	6.42
2490	0.56	2.2	0.6	2.47	0.68	3.03
TA109	0.83	5.39	0.88	7.7	0.88	7.78
**Mean**	0.84	7.61	0.85	8.77	0.87	9.24
**SD**	0.12	3.66	0.12	4.04	0.09	4.62

The mean allele richness (Ar) across the study sites was 10.6 ± 3.9 in Aduku, 12.5 ± 4.4 in Arua, and 11.9 ± 4.5 in Masafu (Supplementary Table S2). The number of genotypes for the K1 allelic family ranged from 14 to 18 in Aduku and Arua, respectively, while for I/C 3D7, it ranged from 19 to 25 in Aduku and Masafu. The most polymorphic microsatellite, 313, exhibited 22 to 28 genotypes in Aduku and Arua. Poly-α genotypes ranged from 15 to 24 in Aduku and Masafu. Microsatellite 383 showed 14 to 20 genotypes in Aduku and Arua. The less polymorphic microsatellite 2490 had 5 genotypes in Aduku and 6 genotypes in Arua and Masafu. Markers such as TA1, TA109, PfPK2, 2490, and *glurp* exhibited 3 genotypes with allele frequencies greater than 10%, with 2490 showing a single genotype with a frequency exceeding 50% (Supplementary Figure S1). The Kruskal-Wallis test revealed no significant differences in the observed number of genotypes, allele richness (Ar), allele frequencies, number of effective alleles (N_e_), or expected heterozygosity (H_e_) across the sites (*p* > 0.05). These results suggest that there is no significant variation in genetic diversity across the three sites, despite the observed differences in allele richness and genotype distribution.

### *P. falciparum* multiplicity of infection (MOI) across three malaria-endemic sites in Uganda

Across all study sites, *msp-1* and the microsatellites PfPK2 and Poly-α exhibited the highest average MOIs, while microsatellite 313, the most diverse marker, generally showed lower MOI. In Aduku, the highest average MOIs for microsatellites were observed for PfPK2, TA1, and Poly-α, with values of 1.77, 1.68, and 1.59, respectively. In Arua, PfPK2 had the highest average MOI of 1.71, followed by TA1 and 383, each with an MOI of 1.59. In Masafu, Poly-α, TA1, and TA109 showed the highest MOIs, with average values of 1.79, 1.68, and 1.58, respectively (Fig. 2). Across all three sites, *glurp* consistently exhibited one of the lowest MOIs. The Kruskal-Wallis test revealed significant differences in MOIs: Poly-α had significantly higher MOIs in Masafu, PfPK2 exhibited significantly higher MOIs in Arua and Aduku, and *msp-1* showed significantly higher MOIs in Arua and Masafu (*P* < 0.05).

Spearman’s rank correlation analysis showed no significant correlation between mean H_e_ and mean MOI (*p* > 0.05).

**Fig. 1:**
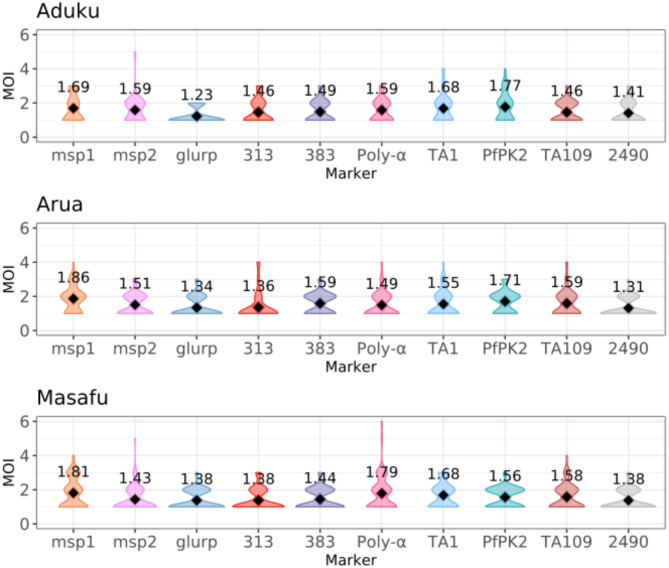
Distribution of the P. falciparum MOI across the three study sites. Each violin plot shows, for a given site, the distribution of the observed MOIs in the samples collected at Day 0. The diamond from each violin plot represents the average MOI whose corresponding value is displayed above.

### Comparison of three algorithms to classify recrudescence across three malaria-endemic sites in Uganda

To classify *P. falciparum* malaria recrudescences, we generated all possible genetic marker combinations by pairing *glurp* and each microsatellite with *msp-1* and *msp-2*, resulting in eight distinct marker combinations. These datasets were then analyzed using three classification algorithms: two match-counting algorithms (≥ 2/3 and 3/3) and a Bayesian algorithm. The goal was to classify each infection as either a recrudescence or a reinfection. Table 2 details the number of recrudescences classified by each marker combination across the different treatment arms. The Wilcoxon signed-rank test revealed that the ≥ 2/3 match-counting algorithm classified significantly more recrudescences than the 3/3 algorithm across both treatment arms (AL and DP) (*P* ≤ 0.05).

**Table 2:** Number of recrudescences classified by the match-counting algorithms. Each row represents one of the eight possible combinations defined by the third marker indicated in the leftmost column.

Third marker	AL treatment arm		DP treatment arm	
	≥ 2/3	3/3	≥ 2/3	3/3
Poly-α	11	2	8	4
PfPK2	14	3	9	4
TA1	10	4	9	2
TA109	12	4	9	2
2490	14	7	8	3
313	12	3	7	3
383	12	2	8	4
*glurp*	11	5	10	4

The columns report the number of recrudescences classified by the ≥ 2/3 and 3/3 algorithms across all three sites for each drug arm. AL = Artemether-Lumefantrine. DP = Dihydroartemisinin-piperaquine.

The Bayesian algorithm was applied to estimate the probability of recrudescence for all samples across eight marker combinations (Supplementary Table S3). Upon visual inspection of the probability distributions, we observed that while there was no clear, definitive cutoff to distinguish recrudescence from reinfection, certain marker combinations exhibited relatively distinct thresholds. Specifically, in the AL arm, combinations of *msp-1* and *msp-2* with *glurp*, *msp-1* and *msp-2* with microsatellite 313, *msp-1* and *msp-2* with microsatellite 383, and *msp-1* and *msp-2* with microsatellite 2490 showed a relatively clear separation in probability distributions. For the DP arm, combinations of *msp-1* and *msp-2* with *glurp*,* msp-1* and *msp-2* with microsatellite Poly-α, *msp-1* and *msp-2* with microsatellite TA109, *msp-1* and *msp-2* with microsatellite PfPK2, and *msp-1* and *msp-2* with microsatellite 2490 also exhibited more discernible probability distributions.

**Fig. 2:**
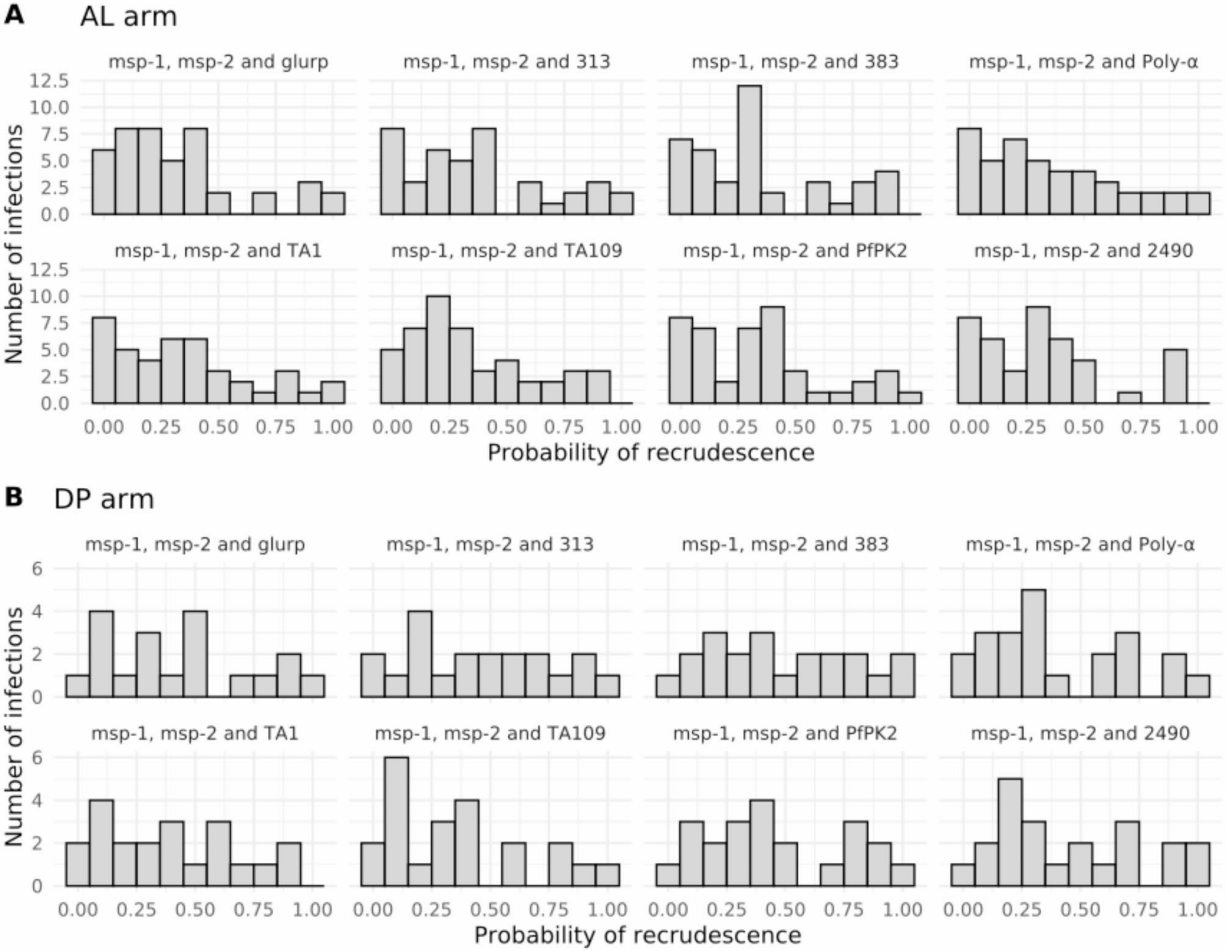
Distribution of the probability of recrudescence estimated with the Bayesian algorithm. Results are presented for the infections in all three study sites in the AL drug arm (**A**) and the DP drug arm (**B**). Each sub-panel displays, for each marker combination, the distribution of probabilities of recrudescence estimated using the algorithm. The heights of the bars correspond to the number of samples with the respective probability.

Based on visual inspection of these distributions, we identified a threshold range between 0.7 and 0.8 as a potential cutoff for classifying recrudescence. These observations suggest that while a clear, universally applicable cutoff was not evident across all combinations, certain marker combinations provided a relatively good distinction, particularly in the AL and DP drug arms (Fig. 2).

A probability cutoff was applied to classify infections as either recrudescence or reinfection. Infections with a probability above the cutoff were classified as recrudescences. The Wilcoxon signed-rank test revealed that the number of recrudescences classified using a 0.7 cutoff was significantly higher than those classified using a 0.8 cutoff (*P* ≤ 0.05) (Table 3).

**Table 3:** Number of recrudescences identified by the Bayesian algorithm. Each row represents one of the eight possible marker combinations defined by the third marker indicated in the leftmost column.

Third marker	AL treatment arm		DP treatment arm	
	0.8	0.7	0.8	0.7
Poly-α	7	13	4	6
PfPK2	8	13	6	10
TA1	7	14	5	6
TA109	6	12	7	9
2490	11	17	5	9
313	10	17	5	9
383	5	13	5	12
*glurp*	7	15	6	8

The remaining columns report the number of recrudescences identified per site using a 0.7 and a 0.8 cutoff for the probability of recrudescence, as well as the total number of recrudescences across all three sites for each drug arm. AL = Artemether-Lumefantrine. DP = Dihydroartemisinin-piperaquine.

We compared the total number of recrudescences classified by each algorithm and marker combination. Results varied widely (Fig. 2), with no clear pattern where a specific marker combination consistently yielded the lowest or highest number of recrudescences.

**Fig. 3:**
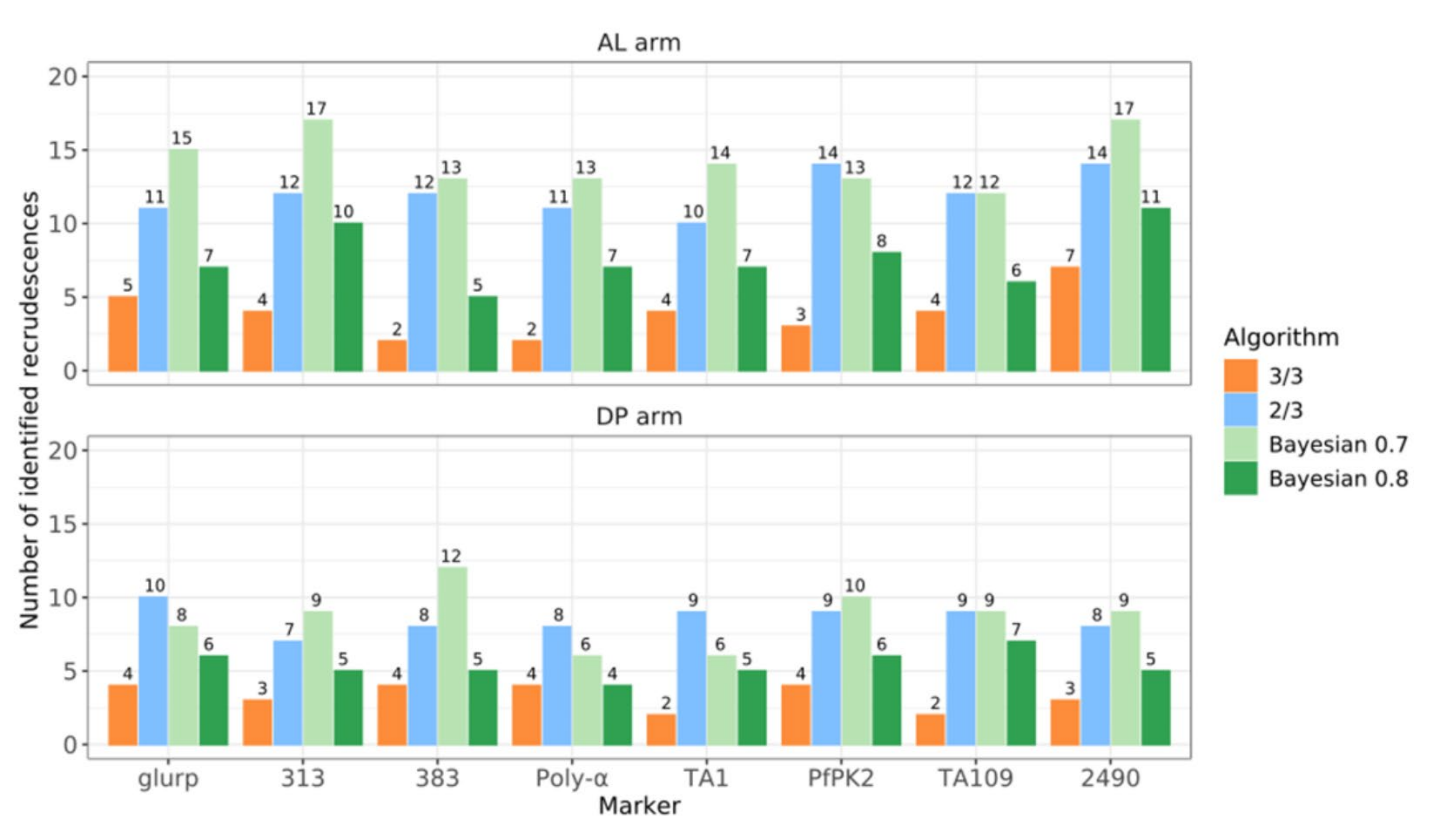
Comparison of the number of recrudescences identified by each algorithm and marker combination. Results are presented for the ≥ 2/3, 3/3, and Bayesian algorithm using cutoffs of 0.7 and 0.8 for the probability of recrudescence, across each marker combination (x-axis). The number of recrudescences identified by each algorithm (y-axis) is illustrated with the colored bars

In general, the 3/3 algorithm was the most conservative, identifying the fewest recrudescences across all marker combinations. For the AL arm, this ranged from two recrudescences with *msp-1*, *msp-2* paired with microsatellites 383 and Poly-α, to seven recrudescences using microsatellite 2490 as third marker. Similarly, for the DP arm, the 3/3 algorithm identified two recrudescences when using microsatellites TA1 or TA104 as the third marker, compared to four recrudescences when using *glurp*, 383, Poly-α, or PfPK2. In contrast, the ≥ 2/3 algorithm identified significantly more recrudescences. For the AL arm, the number ranged from minimum ten recrudescences with microsatellite TA1 to fourteen with PfPK2 and 2490. For the DP arm, it ranged from seven recrudescences with microsatellite 313 to ten with *glurp* (Fig. 3).

The number of recrudescences classified by the Bayesian algorithm varied significantly depending on the probability cutoff used. For the AL arm, on average, nearly twice as many recrudescences were identified with a 0.7 cutoff compared to 0.8, while for the DP arm, the 0.7 cutoff resulted in over 62% more recrudescences than with the 0.8 cutoff. The marker combinations yielding the highest and lowest numbers of recrudescences also varied with the cutoff and drug arm. Specifically, microsatellites 313 and 2490 produced the highest number of recrudescences for both thresholds in the AL arm, while microsatellites TA109 and 383 resulted in the lowest number at the 0.7 and 0.8 thresholds, respectively. In the DP arm, microsatellite 383 led to the most recrudescences at the 0.7 cutoff, while TA109 produced the highest number at 0.8 cutoff. For the same drug arm, the fewest recrudescences were identified using Poly-α and TA1 at the 0.7 cutoff, and Poly-α again at the 0.8 cutoff (Fig. 3).

## Discussion

Accurately distinguishing *P.* *falciparum* recrudescence from reinfection is critical for evaluating antimalarial drug efficacy. The WHO recommends genotyping markers such as *msp-1*, *msp-2*, and highly polymorphic microsatellites like Poly-α, PfPK2, and TA1 for this purpose^10^. However, studies comparing these markers for recrudescence classification, especially in high-transmission areas, are limited. Our study aimed to address this gap by evaluating eight marker combinations and three classification algorithms in recurrent *P. falciparum* malaria infections from a TES assessing AL and DP.

Our study, conducted in areas with high malaria transmission, revealed considerable genetic diversity (mean H_e_ > 0.8) and a mean MOI ranging from 1.23 to 1.86 among *P. falciparum* isolates, consistent with findings from other malaria-endemic regions^17^. This high diversity, shaped by host immunity, ecological factors, and malaria control efforts, complicates malaria control and facilitates the emergence of drug-resistant strains^18–20^.

Microsatellite markers, particularly those with high diversity and MOI, are sensitive in detecting minority clones, making them valuable for distinguishing recrudescence from reinfection^21,22^. Our results showed that while *glurp* exhibited high diversity (H_e_ = 0.86–0.92), its lower MOI (1.23–1.38) resulted in fewer cases of recrudescence being classified. This aligns with previous studies that noted *glurp*’s moderate diversity but lower sensitivity for detecting minority clones^23^. The low MOI of *glurp* suggests reduced sensitivity in identifying minority clones compared to other markers, potentially underestimating recrudescence consequently overestimating drug efficacy^13^. However, some studies have found no significant differences when *glurp* was used instead of microsatellites^24^, though its reduced sensitivity remains a limitation.

Interestingly, markers like *PfPK2* exhibited higher MOIs than expected, contradicting earlier reports of low diversity for this marker^14^. Conversely, markers like 313 and *glurp*, which are more diverse, showed lower MOIs, indicating that high genetic diversity in microsatellites can hinder their ability to detect minority clones, complicating recrudescence classification. While MOI often serves as a proxy for diversity, it does not fully capture a marker’s sensitivity, highlighting the need for further studies to evaluate the utility of diverse microsatellites.

Regarding classification algorithms, the WHO recommends the 3/3 match-counting approach for classifying recrudescence, with the alternative ≥ 2/3 approach suggested for specific settings^10^. Our findings indicate that the 3/3 approach classified significantly fewer recrudescences compared to other algorithms. While this stricter approach increases specificity, it may misclassify true recrudescences as reinfections, particularly in high-transmission areas where genetic diversity and multiple clones are common. In contrast, the ≥ 2/3 approach classified significantly more recrudescences, especially when using microsatellite markers such as 383 or PfPK2 in the AL treatment arm. This suggests that the ≥ 2/3 approach may be more suitable for low- to moderate-transmission areas, although it risks overestimating recrudescence and inflating drug efficacy estimates in high-transmission regions^10^.

We observed variability in the number of recrudescences classified by different marker combinations and algorithms. Combinations of *msp-1*, *msp-2*, and *glurp*, or *msp-1*, *msp-2*, and 2490, identified more recrudescences with both the 3/3 and ≥ 2/3 match-counting algorithms. The low diversity and MOI of 2490 likely account for its higher recrudescence count, while the increased count for *msp-1*, *msp-2*, and *glurp* may be due to the 50 bp bin size used for genotyping, which could group similar genotypes together, leading to overestimation. Additionally, the higher recrudescence count for *glurp* may result from PCR amplification bias, where shorter fragments are preferentially amplified, limiting the detection of minority clones^13,25^.

The Bayesian algorithm performed poorly in our study, with a flat distribution of likelihoods for recrudescence classification. Unlike previous studies from Angola, which reported a bimodal distribution between recrudescences and reinfections^15^, our analysis found that the Bayesian algorithm did not provide useful thresholds for distinguishing between recrudescence and reinfection. This suggests that the genotyped markers lacked sufficient discriminatory power in this context.

In high-transmission areas, microsatellites like Poly-α and 313 have been suggested as useful for distinguishing recrudescence from reinfection^14^. Our study found that markers like PfPK2 and Poly-α, which exhibited higher MOIs, performed better in classifying recrudescence compared to *glurp*. These markers may therefore offer more reliable results in high-transmission areas, supporting their use in such settings. In contrast, markers like *glurp* may still perform adequately in low-transmission regions, where a more lenient algorithm like ≥ 2/3 match-counting could help reduce misclassification of recrudescences as reinfections.

While a standardized approach for PCR correction could improve consistency across studies^10^, a more flexible, adaptive framework that considers local transmission intensity and genetic diversity would enhance recrudescence detection accuracy. Our study suggests that a one-size-fits-all approach is not feasible across diverse malaria transmission environments. Future research should explore adapting PCR correction techniques to different regional settings to improve recrudescence classification and drug efficacy assessments.

Despite these crucial findings, we acknowledge the limitations of our study, particularly regarding the lower discriminatory power of the QIAxcel system, which may have influenced allele calling and recrudescence classification. Higher-resolution techniques, such as deep amplicon sequencing or Applied Biosystems (ABI) capillary electrophoresis, could improve the accuracy of our results. Additionally, the absence of a gold-standard reference method for comparing our computational approaches may have impacted the interpretation of our findings.

In conclusion, our study suggests that PfPK2 and Poly-α are more effective at classifying recrudescence in high-transmission areas and could replace or complement *glurp* in these settings. The findings also highlight the limitations of the Bayesian algorithm in this context. Further validation of these markers in different malaria-endemic regions is necessary to optimize recrudescence classification methods and improve the reliability of TES. Our study underscores the need for flexible, adaptive approaches to PCR correction, taking into account regional transmission intensity and genetic diversity, to enhance recrudescence detection across diverse malaria transmission settings.

## Methods

### Study sites and sample collection

Dried blood spot (DBS) filter paper samples were collected from children aged 6 months to 10 years with recurrent uncomplicated *P. falciparum* malaria, confirmed by positive malaria microscopy. These children were enrolled in a TES assessing the efficacy of AL and DP. The study was conducted from September 2018 to February 2019 at three health centers in Uganda: Aduku Health Centre IV in Kwania district, Northern Uganda; Arua Regional Referral Hospital in Arua district, northwestern Uganda; and, Masafu District Hospital in Busia district, Eastern Uganda. All sites experience perennial malaria transmission with high transmission intensity. A 2018– 2019 malaria indicator survey showed 13% parasite prevalence among children aged 0–59 months in Kwania district, 22% in Arua district, and 21% in Busia district^16^. A total of 179 paired samples were genotyped, comprising 123 paired samples from the AL arm and 56 paired samples from the DP arm (Supplementary Table S1). Further details about the drug efficacy and safety study, are publicly available and described elsewhere^26^.

## Laboratory methods

### DNA extraction

Genomic DNA was extracted from DBS filter paper samples using Chelex 100 Resin (Sigma-Aldrich, USA) as previously described^27^. Briefly, 6 mm discs were punched out from the DBS filter paper into 1.5 mL microcentrifuge tubes containing 1 mL of 1X phosphate-buffered saline (PBS) and incubated at 4 °C overnight. The discs were washed twice in 1 mL PBS and then boiled at 99 °C in 200 µL of 20% Chelex (Sigma-Aldrich, USA) in DNase/RNase-free water. After a final centrifugation step (14,000 × g for 1 min), the extracted DNA was transferred into a labeled 0.6 mL microcentrifuge tube with a 100 µL elution volume and stored at − 20 °C until further use.

### *P. falciparum msp-1*, msp-2 and *glurp* genotyping

Parasite genotyping was conducted at the Infectious Diseases Research Collaboration (IDRC) molecular biology research laboratory in Uganda using nested PCR as previously described^28^ adjusting the PCR reaction mixture to 25 µL. DNA from *P. falciparum* reference strains (3D7, and HB3) was used in each run as positive control^29^. Genotyping was done using nested PCR (Supplementary Table S4). A 3µL sample of the PCR product was then analysed on a 2% agarose gel to confirm the amplification.

The PCR products for the *msp-1* and *msp-2* allele families were analysed by capillary electrophoresis using a Qiaxcel DNA High-Resolution Kit (Qiagen) in the Qiaxcel Advanced system (Qiagen, Hilden, Germany). The analysis followed the AM420 protocol utilising the QX-DNA size marker 50–800 bp (Qiagen, Hilden, Germany) and the QX alignment marker 15 bp/1 kb (Qiagen, Hilden, Germany). The nested PCR products for *glurp* were analysed using the AM420 protocol with the QX DNA size marker 100 bp − 2.5 kb (Qiagen, Hilden, Germany) and the QX alignment marker 15 bp/3 kb (Qiagen, Hilden, Germany). The band sizes were determined with the Qiaxcel Screen Gel software (Version 1.5.0.16, Qiagen, Hilden, Germany) and the height cut off for minority clones was set at 10% for *msp-1* and *msp-2* and 20% for *glurp* of the dominant peak^13^.

### Microsatellite genotyping

Samples were genotyped for the microsatellites TA1, Poly-α, PfPK2, TA109, 2490, 313, 383 at the Centers for Disease Control and Prevention (CDC) Malaria Laboratory in Atlanta, USA. Fragment sizes were then measured using capillary electrophoresis on Applied Biosystems (ABI) 3033 and scored using GeneMarker^®^ V2.6.3, as previously described^26^. For samples that produced more than one peak, the highest peak was defined as the dominant allele. Additional peaks were classified as minor alleles if their peak heights exceeded 200 relative fluorescence units (RFU) and were > 30% of the height of the dominant peak. This threshold was used to identify minor alleles, which may represent clones present at lower frequencies but still contribute to the genetic diversity of the infection. The identification of these additional minor alleles, including third and fourth alleles, was based on the relative peak heights at each microsatellite locus. Peaks that met these criteria were recorded as distinct alleles.

Genotyped samples were considered successful if they amplified on all markers (*msp-1*, *msp-2*,* glurp*, and microsatellites) on both day 0 and the day of failure. Samples that did not amplify on all markers were excluded from the final analysis.

### Data analysis

To further refine the identified fragment sizes and determine the final alleles for each marker, we grouped the fragments based on predefined bin sizes. For the *msp-1* and *msp-2* allelic families, we used a bin size of 10; for *glurp*, a bin size of 50; for the double repeat microsatellites 313 and 383, a bin size of 1; and for the triple repeats microsatellites TA1, Poly-α, PfPK2, TA109, and 2490, a bin size of 2. Fragments with length differences within the specified bin sizes were grouped together. The grouping operation started with an offset identified to achieve the highest overlap between the final (theoretical) alleles and observed fragment sizes.

*Genetic diversity*. For each site, we determined the genetic diversity of the genotyped markers by calculating both the mean number of genotypes, allele richness (Ar), allele frequency and the number of effective alleles (N_e_) at each locus using GENALEX 6.5 software^30^. Expected heterozygosity (H_e_) which is the probability that two randomly selected individuals from a population will carry distinct alleles at each marker locus, was calculated using ARLEQUIN software version 3.11^31^ with the following formula:

$$H_{e} {{ = [}}n{{/(}}n - {{1)][1}} - \sum {p_{i} ^{2} } {{],}}$$

where ‘*n*’ and ‘*p*_*i*_’ represent the number of isolates analysed and the frequency of the allele in a given population, respectively. H_e_ values range between 0 and 1 from no genetic diversity to high genetic diversity, respectively as previously described by Anderson et *al.*^18^. Parasite isolates with higher values of H_e_ and N_e_​ are indicative of greater genetic diversity within the population^32,33^.

*P. falciparum* MOI, defined as the number of distinct parasite clones simultaneously infecting an individual^34^, was estimated for each sample at day 0 (day of the primary infection) under the different drug arms and across the three sites. For each site and marker, we then determined the mean MOI by identifying the highest number of alleles observed across any of the microsatellite markers used in the analysis. This maximum allele count was considered the MOI for that particular infection.

*Recrudescence and reinfection classification*. Three algorithms were applied to the identified alleles from all the samples, namely the 3/3 match counting algorithm, ≥ 2/3 match counting algorithm, and a Bayesian approach^10,15^. These algorithms were applied to several datasets, each combining different genotyped microsatellites as well as *glurp* with *msp-1* and *msp-2*. The 3/3 method classified an infection as recrudescence if one or more alleles occurred at all three genotyped markers on both day 0 and the day of the recurrent malaria infection. In contrast, a ≥ 2/3 match counting algorithm classified an infection as a recrudescence if one or more alleles were present in at least two of the three genotyped markers on both day 0 and the recurrence day. The Bayesian algorithm utilized allele information from all samples to estimate the probability of recrudescence for each case. A low probability indicated a greater likelihood of reinfection, whereas a probability closer to 1 suggested a high likelihood of recrudescence^15^. This approach highlights how different probability thresholds can influence the sensitivity (true positive rate) and specificity (true negative rate) of the algorithm. Specifically, a lower threshold may lead to the identification of more recrudescences, thereby enhancing sensitivity, while a higher threshold can improve specificity by reducing false positives. To classify recrudescence versus reinfection, probability thresholds of 0.7 and 0.8 were applied sequentially.

*Statistical analysis*

Genetic diversity and MOI across sites were compared using the Kruskal-Wallis test, while correlation between mean H_e_ and mean MOI was assessed using Spearman’s rank correlation. The results of all three algorithms (3/3 match-counting, ≥ 2/3 match-counting, and Bayesian) were then compared using the Wilcoxon signed-rank test to assess their effectiveness in identifying recrudescences based on the hypothesis that there would be no difference in the number of recrudescences classified by the ≥ 2/3 and 3/3 algorithms for each treatment arm (both AL and DP). All the analyses were conducted using STATA version 17 (Stata Corporation, College Station, TX), with a statistical significance set at *P* < 0.05.

## Electronic supplementary material

Below is the link to the electronic supplementary material.

Supplementary Material 1

Supplementary Material 2

Supplementary Material 3

Supplementary Material 4

Supplementary Material 5

## Data Availability

The datasets generated and/or analysed in this study are available from the corresponding author upon reasonable request. All the analysis code used in the study and for generating all the figures is available at: https://github.com/SwissTPH/genotyping_comparison_Uganda/tree/main.
