# Supplementary material for: Assessment of different genotyping markers and algorithms for distinguishing Plasmodium falciparum recrudescence from reinfection in Uganda
Source: Sci Rep. 2025 Feb 5;15:4375. doi: 10.1038/s41598-025-88892-7 (PMC11799330; doi:10.1038/s41598-025-88892-7)
Supplement: Supplementary file 1 — Supplementary Material 1 [file 41598_2025_88892_MOESM1_ESM.doc]

**Supplementary table S1: Number of samples genotyped per treatment arm**

| Drug | AL Treatment arm | | | DP Treatment arm | | |  |
| --- | --- | --- | --- | --- | --- | --- | --- |
| Study site | Aduku | Arua | Masafu | Aduku | Arua | Masafu | Total |
| Number retrieved | 60 | 78 | 120 | 24 | 40 | 50 | 372 (186 pairs) |
| Number genotyped | 60 | 78 | 108 | 24 | 40 | 48 | 358 (179 pairs) |
| Number analyzed | 56 | 78 | 106 | 22 | 40 | 46 | 348 (174 pairs) |
