# Supplementary material for: Assessment of different genotyping markers and algorithms for distinguishing Plasmodium falciparum recrudescence from reinfection in Uganda
Source: Sci Rep. 2025 Feb 5;15:4375. doi: 10.1038/s41598-025-88892-7 (PMC11799330; doi:10.1038/s41598-025-88892-7)
Supplement: Supplementary file 2 — Supplementary Material 2 [file 41598_2025_88892_MOESM2_ESM.doc]

**Supplementary table S2**. Allele richness of *P. falciparum* across sites

| **Marker** | **Aduku** | **Arua** | **Masafu** |
| --- | --- | --- | --- |
| K1 | 12.8 | 15.2 | 14.6 |
| MAD20 | 7.4 | 14.8 | 11.3 |
| RO33 | 5.2 | 5.4 | 5.4 |
| IC/3D7 | 17.3 | 19.2 | 11.8 |
| FC 27 | 12.3 | 13.2 | 12.1 |
| *Glurp* | 7.6 | 8.2 | 14.2 |
| C2M34–313 | 17.5 | 18.4 | 21.0 |
| C3M69–383 | 9.0 | 11.8 | 11.0 |
| Poly-ɑ | 12.9 | 14.4 | 15.4 |
| TA1 | 12.0 | 15.8 | 15.9 |
| PfPK2 | 9.7 | 11.6 | 9.3 |
| 2490 | 5.6 | 5.9 | 4.9 |
| TA109 | 9.1 | 9.1 | 7.5 |
| **Mean** | 10.6 | 12.5 | 11.9 |
| **SD** | 3.9 | 4.4 | 4.5 |
