# Supplementary material for: Assessment of different genotyping markers and algorithms for distinguishing Plasmodium falciparum recrudescence from reinfection in Uganda
Source: Sci Rep. 2025 Feb 5;15:4375. doi: 10.1038/s41598-025-88892-7 (PMC11799330; doi:10.1038/s41598-025-88892-7)
Supplement: Supplementary file 3 — Supplementary Material 3 [file 41598_2025_88892_MOESM3_ESM.doc]

**Supplementary figure S1**: Number of genotypes and allele frequencies of the genotyped markers


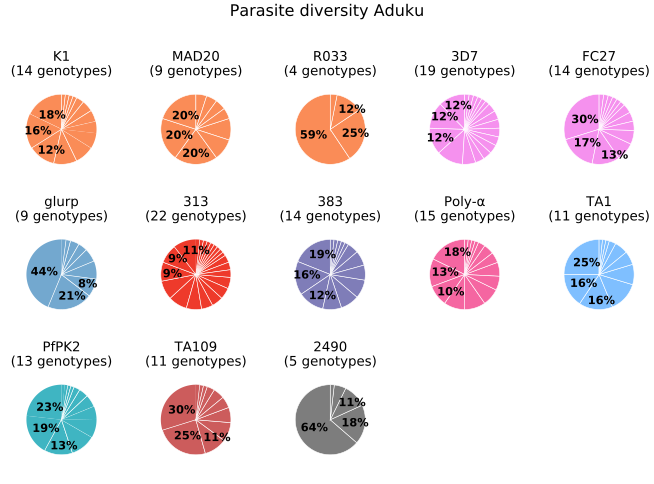


*
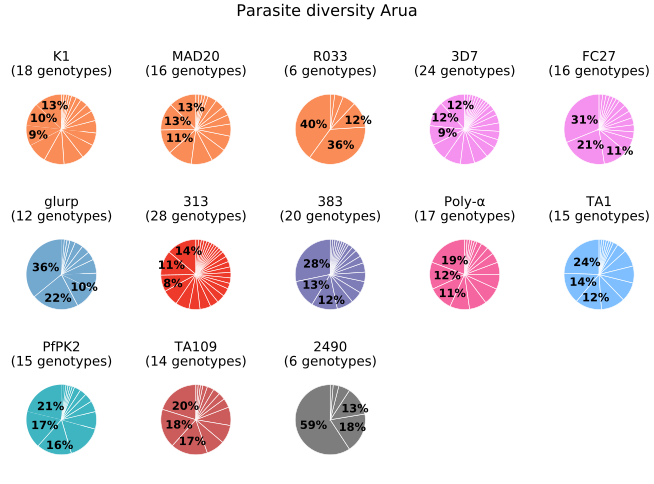
*

*
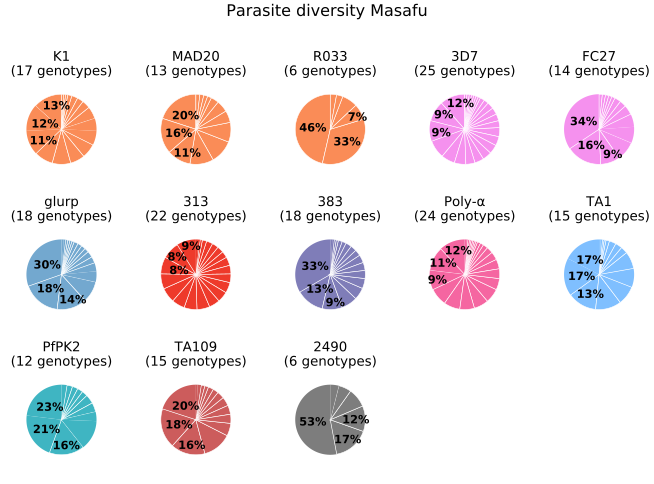
*

***Figure S3:*** *Parasite diversity observed in samples from the three study sites: Aduku, Arua, and Masafu. Each pie chart represents the distribution of observed genotypes for a specific genetic marker at day 0. The total number of genotypes for each marker is noted below its name. The percentages for the three most frequent genotypes are displayed on the corresponding slices of the pie charts, calculated as the proportion of samples with each genotype relative to the total number of samples.*
