# Supplementary material for: Assessment of different genotyping markers and algorithms for distinguishing Plasmodium falciparum recrudescence from reinfection in Uganda
Source: Sci Rep. 2025 Feb 5;15:4375. doi: 10.1038/s41598-025-88892-7 (PMC11799330; doi:10.1038/s41598-025-88892-7)
Supplement: Supplementary file 4 — Supplementary Material 4 [file 41598_2025_88892_MOESM4_ESM.doc]

**Supplementary table S3**. Recrudescence outcome based on the cumulative Bayesian probabilities.

| **Third marker** | **AL treatment arm** | **DP Treatment arm** |
| --- | --- | --- |
| Poly-α | 32 | 18 |
| PfPK2 | 36 | 20 |
| TA1 | 34 | 18 |
| TA109 | 33 | 16 |
| 2490 | 34 | 17 |
| 313 | 35 | 18 |
| 383 | 40 | 19 |
| *glurp* | 33 | 16 |
