# Supplementary material for: Assessment of different genotyping markers and algorithms for distinguishing Plasmodium falciparum recrudescence from reinfection in Uganda
Source: Sci Rep. 2025 Feb 5;15:4375. doi: 10.1038/s41598-025-88892-7 (PMC11799330; doi:10.1038/s41598-025-88892-7)
Supplement: Supplementary file 5 — Supplementary Material 5 [file 41598_2025_88892_MOESM5_ESM.doc]

**Supplementary table S4:** *msp-1*, *msp-2* and *glurp* genotyping

**Master mix** components and preparation

| **Component** | **Stock concentration** | **Final concentration** | **Volume for one reaction (µL)** |
| --- | --- | --- | --- |
| PCR water | …….. | …… | 16.8 |
| Forward primer | 1010 µM | 0.2 µM | 0.5 |
| Reverse primer | 10 µM | 0.2 µM | 0.5 |
| Buffer | 10X | 1X | 2.5 |
| dNTPs (2 mM), 0.75 MgCl_2_ (50mM), |  | 200 µM | 2.5 |
| Taq Polymerase | 5 U/µL | 1.5 mM | 0.2 |
| Template DNA |  |  | 2 |
| Final Volume |  |  | 25 |

**PCR conditions**

Msp-1

| **Stages** | **Steps** | **Primary round** | | | **Nested round** | | |
| --- | --- | --- | --- | --- | --- | --- | --- |
|  |  | **Temperature (^0^C)** | **Time** | **Cycles** | **Temperature (^0^C)** | **Time** | **Cycles** |
| Initial denaturation |  | 95 | 5 min | 1 | 95 | 2 min | 1 |
| 2. PCR | 1. Denaturation | 94 | 1 min | 25 | 94 |  | 30 |
|  | 2. Annealing | 61 | 45 sec |  | 61 | 30 sec |  |
|  | 3. Extension | 72 | 1.5 min |  | 72 | 1 min |  |
| 3. Final elongation & Hold | 1. Final elongation | 72 | 5 min | 1 | 72 | 5 min |  |
|  | 2. Hold | 4 | ∞ |  | 4 | ∞ |  |

Msp-2

| **Stages** | **Steps** | **Primary round** | | | **Nested round** | | |
| --- | --- | --- | --- | --- | --- | --- | --- |
|  |  | **Temperature (^0^C)** | **Time** | **Cycles** | **Temperature (^0^C)** | **Time** | **Cycles** |
| Initial denaturation |  | 94 | 5 min | 1 | 94 | 2 min | 1 |
| 2. PCR | 1. Denaturation | 94 | 1.5 min | 40 | 94 | 1.5 | 30 |
|  | 2. Annealing | 55 | 45 sec |  | 55 | 45 sec |  |
|  | 3. Extension | 72 | 1.5 min |  | 72 | 1.5 min |  |
| 3. Final elongation & Hold | Final elongation | 72 | 10 min | 1 | 72 | 10 min |  |
|  | 2. Hold | 4 | ∞ |  | 4 | ∞ |  |

Glurp

| **Stages** | **Steps** | **Primary round** | | | **Nested round** | | |
| --- | --- | --- | --- | --- | --- | --- | --- |
|  |  | **Temperature (^0^C)** | **Time** | **Cycles** | **Temperature (^0^C)** | **Time** | **Cycles** |
| Initial denaturation |  | 95 | 5 min | 1 | 95 | 2 min | 1 |
| 2. PCR | 1. Denaturation | 94 | 1 min | 25 | 94 | 1 min | 30 |
|  | 2. Annealing | 58 | 2 min |  | 58 | 2 min |  |
|  | 3. Extension | 72 | 2 min |  | 72 | 2 min |  |
| 3. Final elongation & Hold | 1. Final elongation | 72 | 10 min | 1 | 72 | 10 min |  |
|  | 2. Hold | 4 | ∞ |  | 4 | ∞ |  |
